# Adolescents’ voices on self-engagement in mental health treatment: a scoping review

**DOI:** 10.1007/s00787-024-02425-7

**Published:** 2024-03-27

**Authors:** Nina Therese Øversveen Svamo, Sigrid Helene Kjørven Haug, Valerie DeMarinis, Urd Hertzberg

**Affiliations:** 1 Research Center for Existential Health, Innlandet Hospital Trust, Brumunddal, Norway; 2 Inland Norway University of Applied Sciences, Lillehammer, Norway; 3 Inland Norway University of Applied Sciences, Elverum, Norway; 4 Department of Public Health and Clinical Medicine, Umeå University, Umeå, Sweden; 5 Medical Library, Innlandet Hospital Trust, Brumunddal, Norway

**Keywords:** Adolescents, Mental health treatment, Person-centered care, Self-engagement, Scoping review

## Abstract

Introduction: According to the United Nations Convention on the Rights of the Child, adolescents’ involvement in their healthcare is a fundamental right, and self-engagement in mental health treatment is vital for realizing their potential within person-centered care (PCC). Research exists that highlights barriers to involving adolescents in their care decisions. However, research on adolescents’ own voices about self-engagement in mental health treatment has been scarce. This scoping review aimed to examine and summarize current knowledge on adolescents’ voices regarding self-engagement in mental health treatment. Method: The review followed the scoping methodology of Arksey and O’Malley from 2005, updated by Levac and colleagues in 2010, involving five stages: (1) identifying the research question, (2) identifying relevant studies, (3) study selection, (4) charting the data, and (5) collating, summarizing and reporting the results. Results: Nineteen studies were included. The following themes on adolescents` voices regarding self-engagement in mental health treatment were identified: (1) the therapeutic alliance, (2) the need for active engagement in treatment, (3) different experiences due to time of data collection, (4) treatment context and healthcare system, and (5) adolescent-caregiver interaction. Conclusion: Adolescents’ understanding of self-engagement was multilevel and comprehensive, including individual, contextual and relational factors. A strong therapeutic alliance with healthcare providers, and a need to be actively engaged in treatment were highlighted. To succeed in strengthening PCC in mental healthcare for adolescents, health professionals must take this complex understanding into consideration, as treatment without adolescents` self-engagement may worsen their clinical outcomes. Future research should explore specific PCC interventions and incorporate diverse methodologies in various clinical contexts. Additionally, insights from healthcare providers and caregivers on self-engagement in mental health treatment will complement these findings.

**Supplementary Information:**

The online version contains supplementary material available at 10.1007/s00787-024-02425-7.

## Introduction

Person-centered care (PCC) is a holistic approach to deliver respectful and individualized care [1], based on values such as respect, reciprocity, mutuality, and self-determination [2]. Research shows that adolescents emphasize the importance of their relationship with their healthcare provider (HCP) [3–5]. Among the qualities of the HCP most valued by adolescents are being a good listener and treating them like people, not patients [3–5]. This is in line with adolescents’ expectations for the healthcare system [6]: to be treated with respect and acceptance in a professional manner, with good communication.

The World Health Organization defines adolescents as individuals aged 10–19 years, and young people as individuals younger than 25 years [7]. The United Nations Convention on the Rights of the Child [8] gives adolescents the right to be involved in decisions that affect their healthcare. In addition, the World Health Organization (WHO) emphasizes the value of “adolescent-friendly” healthcare as an approach to adapt health services to adolescent needs [9]. The WHO proposes that self-engagement in mental health treatment is important for adolescents to achieve their potential and to live fulfilling lives as adults [10]. A scoping review by Hawke and colleagues [4] found that integrating adolescents’ voices, in the form of self-engagement, was a key ingredient in adolescent-friendly treatment, which calls for adolescent input to be heard at the organizational and policy levels in terms of both treatment and the social environment. This is in line with the person-centered focus prevalent in healthcare in Norway and other Scandinavian countries [11]. The Scandinavian countries are therefore of particular interest for this article.

There are various operational definitions of self-engagement in treatment, including patient attendance, participation in treatment, a therapeutic alliance, and treatment retention/attrition [12]. Successful self-engagement in treatment depends on a combination of systemic and structural factors such as inclusive services, convenience of location, flexible hours, and continuity of care [4, 13, 14]. A trusting relationship with the HCP should involve appropriate communication and counseling skills [4, 6, 13] and the inclusion of family members in treatment decision-making [4, 15]. Self-engagement in mental health treatment has been associated with positive health outcomes, improved patient-provider communication, better healthcare quality, and decreased costs [16]. However, mental healthcare services have been slow to respond to adolescents’ recommendations, despite knowledge of the significance of their voices [14, 15, 17–20].

Providing a space and a process for adolescents’ voices to be heard as an integral part of planning and conducting the treatment process is dependent not only on a philosophy of PCC that understands the value and necessity of this, but also on the testing of available methods and processes for engaging and integrating adolescents’ thoughts, questions, concerns and perceptions into the treatment process. As indicated in the systematic review by Radez and colleagues [17] on barriers and facilitators of treatment among children and adolescents, they noted the difficulty and complexity of engaging adolescents in treatment. Four themes were identified: individual factors, such as limited knowledge of mental health problems; social factors, such as embarrassment and perceived stigma; perceptions of the relationship between adolescents and HCPs, such as issues of confidentiality; and structural factors [17]. In their literature review of barriers and facilitators around involving adolescents in a mental health setting, Gondek and colleagues [15] named limited resources, lack of information about available services, extensive and inflexible policies and regulations, and inflexible treatment provision as major barriers to delivery of PCC. These barriers point to the need for examining policies and provisions for addressing such barriers and challenges within the structure of the healthcare context and their contributions to providing sub-optimal PCC.

This scoping review focused on addressing this research-to-practice gap. Initially, the review aimed to conduct a broad exploration of the existing literature on PCC and adolescents in mental health treatment in terms of the volume, nature, and characteristics of the primary research [21]. The scope of the review was then narrowed down to the voices of adolescents in mental health treatment, and finally limited to examining studies involving their self-engagement. Hence, the final aim was to describe adolescents’ overall experiences of self-engagement in mental health treatment and to identify important factors that facilitate self-engagement in this context. The following research question was developed: *What are the voices of adolescents saying about self-engagement in mental health treatment?*

## Method

This review was based on an adaption of the methodological framework described by Arksey and O’Malley [21] and further enhanced by Levac and colleagues [22]. The reporting guidelines of the Preferred Reporting Items for Systematic Reviews and Meta-analysis extension for Scoping Reviews (PRISMA-ScR) was used [23]. This framework followed five stages: (1) identifying the research question, (2) identifying relevant studies, (3) study selection, (4) charting the data, and (5) collating, summarizing and reporting the results.

It is important to note that the entire research team was involved in the process. The team encompasses various multidisciplinary competencies, including clinical psychology, mental health and clinical care, milieu therapy for children and adolescents in psychiatry, and library and information science. The diversity of competencies was crucial for the comprehensive exploration of the topic and to avoid bias associated with expertise in a single area.

### Stage 1: identifying the research question

**Fig. 1:**
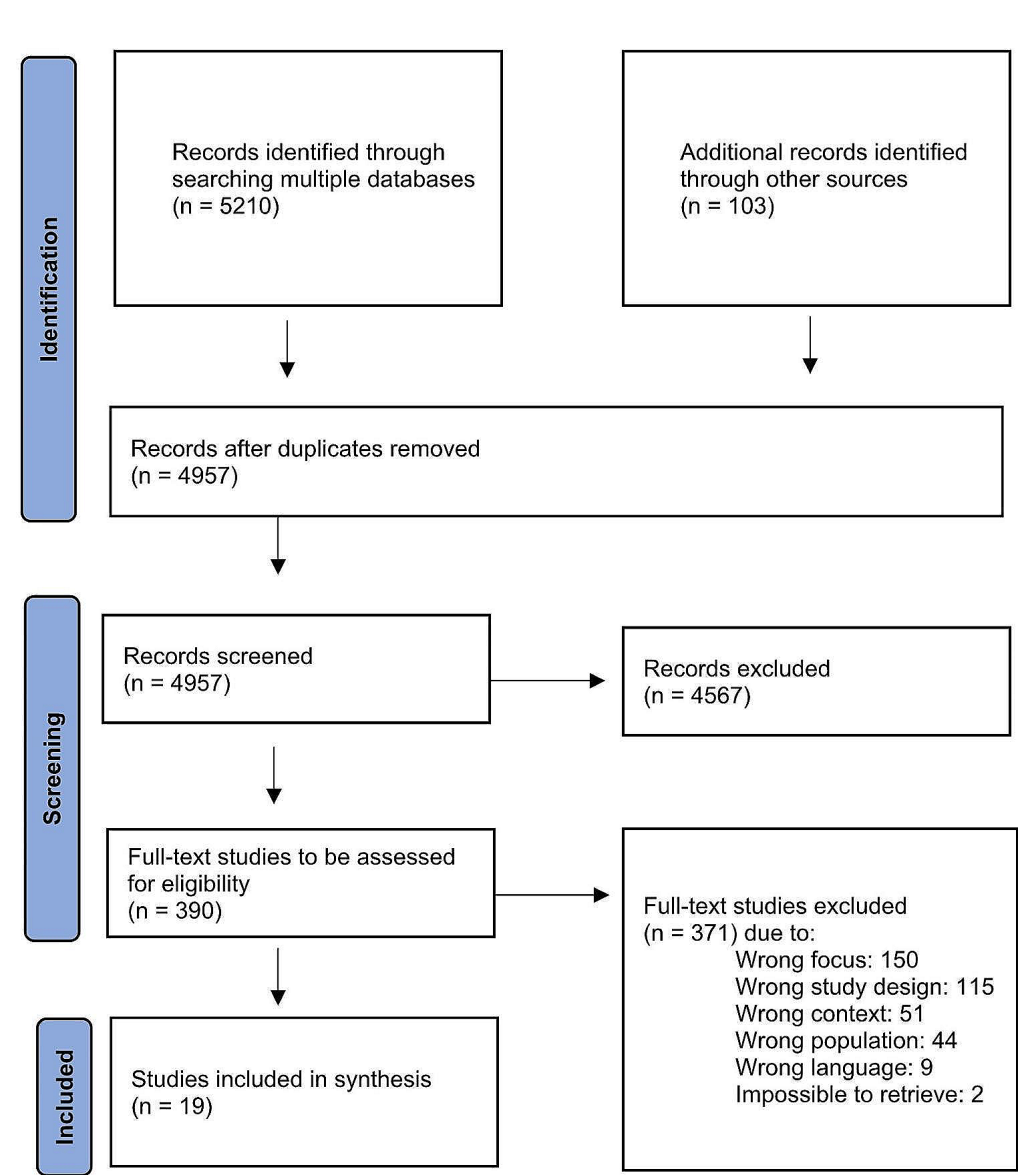
PRISMA flow diagram of the identification and selection of studies

The process of identifying the research question was conducted in three phases (A, B, and C). In phase A, a broad inquiry was initiated in order to identify the existing literature in the field of PCC and adolescents aged 12–18 years receiving treatment in mental healthcare. This identification and selection process is seen in the PRISMA flow diagram, Fig. 1, showing that 4957 studies were included. In phase B, the screening involved reducing the 4957 studies to 390, as also presented in the PRISMA flow diagram. The reduction process implied a narrowing of scope through the development of inclusion and exclusion criteria. In this process, the following tentative research question was formulated: *What are the voices of adolescents saying is important for them in mental health treatment?* Finally, in phase C the research question was revised into its final form focusing on adolescents’ self-engagement in mental health treatment. This research question led to the exclusion of 371 studies, as shown in the diagram.

### Stage 2: identifying the relevant studies

#### Search method/search strategy

A comprehensive search strategy was developed using the PICO framework for converting the research question into searchable elements. While PICO usually defines the relevant population, intervention, comparison and outcome, the search was not constrained to specific comparisons, outcomes or types of study, due to the broad purpose of the review. The population consisted of children and adolescents within the context of mental health institutions, combining search terms for child and adolescent psychology or psychiatry with search terms for health institutions. The intervention consisted of a broadly defined set of search terms, including “patient-centered care”, “person-centered care”, “shared decision making”, “patient-clinician communication”, “personalized medicine”, “patient participation”, “patient customization”, “patient preference” and “professional-patient relationship” (see Supplementary Material1). Searches were conducted by two medical librarians in cooperation with the research group, and peer reviewed by an additional medical librarian. Four international databases were searched (Ovid MEDLINE, Ovid APA PsycInfo, Ovid Embase and Ebsco CINAHL), as were seven Scandinavian databases (Norart, Svemed+, Publicera.se, Oria, Idunn, Swepub and Tidsskrift.dk) because of the relative similarity of the healthcare systems in Scandinavia. Gray literature was sought in Embase, NIH RePORTER, ClinicalTrials.gov, WHO ICTRP, OAIster, NARCIS (Netherlands), Open Grey and RIAN (Pathways to Irish Research). Searching gray literature reduces the risk of bias, and compensates to some extent for the time lag between research and publication, as it includes searching non-published research [24]. Final searches took place in February 2022. Complete search strategies for all international and Scandinavian databases, as well as search and coverage dates for sources of gray literature, can be found in Supplementary Material1.

Two web-based tools were used to further identify studies through citation trails from relevant studies. Using citations as a supplement to a text-based strategy can partly compensate for limitations such as synonymy, homonymy, and inconsistent or incomplete keyword indexing [25]. Belter [25] recommends identifying papers frequently co-cited by or co-citing well-known, relevant studies. Nineteen studies were chosen as “seed articles” (see Supplementary Material1). Citation Gecko (using data from OpenCitations, Crossref and Microsoft Academic) revealed connections between seed articles, and new studies were included for screening if they cited, or were cited by, two or more of the chosen seed papers. The search tool Co-Cites shows studies being co-cited with a specific paper, based on the 100 most recent citations from NIH Open Citation Collection. New studies co-cited three or more times with one of the seed papers were included. This endeavor found 98 papers not identified through the text-based search strategy. In addition, five results were included from a manual review of reference lists of all nineteen seed articles, leaving 103 additional new studies for screening.

#### Eligibility criteria

Inclusion and exclusion criteria were established using an iterative process [22] throughout the three phases presented in Stage 1. According to common practice when conducting a scoping review, eligibility criteria were partly developed post hoc with increased familiarity with the topic [21]. The authors made efforts to balance the scope, breadth and comprehensiveness of the study [22]. The inclusion and exclusion criteria for the final sample are shown in Table 1.

**Table 1:** Inclusion and exclusion criteria

Inclusion criteria	Exclusion criteria
• Adolescents aged 12–18 years, regardless of the type of mental disorder, who receive mental health care and treatment • Studies reporting on adolescents’ voices and views, studies reporting on adolescents’ voices about self-engagement • English or Scandinavian languages • Qualitative design • Mixed methods if the article focused on the qualitative phase • Empirical studies • Original data collection	• Under 12 years or over 18 years • Outcomes which are not adolescent-focused (e.g., focused on parents, family or health professionals, or adult-focused or mainly adult-focused interventions) • Outcomes focused on specific themes (e.g., digital tools, substance use or pregnancy) • Studies from developing countries where the healthcare system is more fragmented • Non-English or non-Scandinavian languages • Quantitative design • Non-empirical study (e.g., editorial, conference abstract, dissertation abstract, books, study protocols) • Non-original data collection (e.g., systematic or scoping review)

### Stage 3: selecting the studies—identifying relevant studies

A flow diagram (Fig. 1) shows the identification and selection process, as recommended by Tricco and colleagues [23]. A total of 5210 studies were retrieved from the initial database search. An additional 103 studies were identified through citation trails either manually or using citation tracking tools. Results were deduplicated using Bramer’s method [26], and duplicates were removed (*n* = 356). Following this, 4957 studies remained. To ensure transparency [22] in the selection process, three researchers (NTØS, SHKH and VDM) were involved in the screening. Titles and abstracts were reviewed in accordance with the eligibility criteria by two authors (NTØS and SHKH), using Rayyan [27]. Exclusion of 4567 studies was based on phases A and B. Studies that were excluded were not on mental health treatment, or focused on caregivers’ or providers’ preferences, needs and outcomes regarding treatment more than those of adolescents. This left 390 studies for review based on the full-text version. At this stage, exclusion of studies was due to the following reasons: wrong focus based on the final phase C (e.g. the adolescents’ focus was on ethical challenges, the implementation and effects of various tools such as motivational interviewing or digital tools), wrong study design, wrong context, wrong population (e.g., adult or pediatric population), wrong language (language that was not English or Scandinavian) or impossible to retrieve. The third researcher (VDM) checked whether the inclusion and exclusion criteria applied to the 390 studies. Uncertainties or disagreements were discussed until agreement was reached, and 371 studies were rejected as not meeting the eligibility criteria or not fitting the research question [21]. Finally, 19 studies were included.

### Stage 4: charting the data

The research group developed a data charting form containing the data to extract from the studies [22]. To identify key concepts, information from selected studies was charted. The results of the data charting process are shown in Table 2.

### Stage 5: collating, summarizing and reporting the results

The findings from the 19 included articles were analyzed with inductive content analysis as described by Elo and Kyngäs [28]. The analysis included the following five steps:

1. Open coding: The analysis process started with gaining a sense of the entire data set. All the answers of the adolescents were incorporated to a table. Headings (keywords) and notes was written down while the text was read to describe all aspects of the written material.
2. Coding sheets: Headings were listed on a coding sheet and categories were freely created.
3. Grouping: The categories were grouped together into higher order headings by combining categories with similar content.
4. Categorization: In this step, similarities and differences in the grouping were identified and divided into categories of main and sub-themes. The categorization was discussed within the research group until consensus was reached.
5. Abstraction: In the final step, the main and sub-themes were grouped into higher-order categories by collapsing those that were similar. The final themes were checked with the initial coding and grouping processes for consistency. The results are presented in Table 3 and thematically summarized in the Results section.

#### Critical appraisal

Although critical appraisal is not a compulsory measure in a scoping review [21], there has been criticism of this [22]. Worrall-Davies and Marino‐Francis [18] report that studies on adolescents’ views are often of poor methodological quality. Therefore, we decided to report on the quality of the included studies. The results were used to depict the general adequacy (or rigor) of studies described in the sample and to enhance our examination of the relationship between the methods and usefulness of the findings. Authors (NTØS, SHKH) independently appraised the final studies for their methodological quality using the tool Critical Appraisal Skills Programme (CASP) [29] for assessing risk of bias in qualitative studies. Disagreements were discussed until consensus was reached. All studies satisfied the first two criteria (clear statement of the aims and appropriate methodology). No studies were excluded based on the CASP results. An overview of these results is found in Supplementary Material 2.

## Results

Results of the numbers of studies at each stage of the selection process are shown in the PRISMA flow chart (Fig. 1). Nineteen studies were included in this review. An overview of year, country, sample size, age range of adolescents, type of data collection, and data collection period of the included studies is presented in Table 2. The results of the thematic findings are shown in Table 3.

**Table 2:** Overview of included studies (*N* = 19)

Study number	First author	Year	Country	Study setting	Sample size and age range ( ) of adolescents	Type of data collection	Data collection period
1.	Armstrong et al.	2019	Canada	Outpatient	*N* = 20 (16–26)	Semi-structured interviews	During treatment
2.	Buston	2002	Scotland	Outpatient	*N* = 32 (14–20)	Semi-structured interviews	During treatment
3.	Byczkowski et al.	2010	United States	Outpatient	*N* = 170 (12–17)	Semi-structured telephone interviews	During treatment
4.	Coates	2016	Australia	Outpatient	*N* = 17 (16–24)	Semi-structured telephone interviews	After treatment
5.	Coyne et al.	2015	Ireland	Outpatient	*N* = 15 (11–17)	Combination of focus group and semi-structured interviews	During treatment
6.	Davison et al.	2017	England	Outpatient	*N* = 17 interview *N* = 34 self- report (12–18)	Combination of self-report and semi-structured interviews	Combination of during and after treatment
7.	Grealish et al.	2013	United Kingdom*	Outpatient	*N* = 9 (14–18)	Semi-structured interviews	During treatment
8.	Harper et al.	2014	England	Outpatient	*N* = 10 (16–18)	Semi-structured interviews	After treatment
9.	Hart et al.	2005	England	Outpatient	*N* = 27 (11–18)	Combination of focus group and semi-structured interviews	During treatment
10.	Hayes et al.	2020	Australia	Inpatient	*N* = 16 (12–22)	Semi-structured interviews	Combination of at admission, during treatment and at the end of treatment
11.	Jones et al.	2017	England	Outpatient	*N* = 10 (16–18)	Semi-structured interviews	During treatment
12.	LeFrançois	2008	United Kingdom*	Inpatient	N = not specified (11–18)	Multi-method ethnographic field study	During treatment
13.	McCann and Lubman	2012	Australia	Outpatient	*N* = 26 (16–25)	Semi-structured interviews	During treatment
14.	Midgley et al.	2016	England	Outpatient	*N* = 77 (11–17)	Semi-structured interviews	First attendance
15.	Munford and Sanders	2016	New Zealand	Outpatient	*N* = 109 (13–17)	Combination of self-report and semi-structured interviews	After treatment
16.	Persson et al.	2017	Sweden	Outpatient	*N* = 7 focus group *N* = 106 self- report (11–17)	Combination of focus group and self-report	During treatment
17.	Ronzoni and Dogra	2012	England	Outpatient	*N* = 60 (6–18)	Self-report with two open-ended questions	First attendance
18.	Salamone-Violi et al.	2015	Australia	Inpatient	*N* = 11 (15–17)	Semi-structured interviews	First attendance, during treatment, at the end of treatment
19.	Stafford et al.	2016	United Kingdom*	Outpatient	*N* = 28 (6–17)	Video recordings of semi-structured interviews	First attendance

* The study did not specify which country in the United Kingdom

**Table 3:** Main themes and sub-themes from the included studies related to adolescent self-engagement in mental health treatment

Theme	Theme 1 Therapeutic alliance	Theme 2 Need for active engagement in treatment	Theme 3 Different experiences due to time of data collection	Theme 4 Treatment context and healthcare system	Theme 5 Adolescent-caregiver interaction
Sub- themes	Characteristics of the HCP Qualities in the relationship Transparency in the treatment process	Actively engaged in all stages of treatment Be recognized as experts Sensitive to mental health stigma	Not knowing what to expect Change in perspectives Uncertainty about the future	Homely environment Access to treatment Daily structure and routines	Trust and confidentiality Different levels of caregiver involvement Caregiver support
Study numbers in which theme emerged	1–19	1–19	1–19	1,2,3,5,6,7,8, 10,12,13,15,16, 18	2,3,4,5,7,8,9, 10,17,18

### Theme 1: the therapeutic alliance (*n* = 19)

All the studies addressed the therapeutic alliance as a key factor in promoting self-engagement in treatment, recognizing that adolescents valued an understanding and supportive alliance between themselves and the HCP.

Key HCP characteristics identified by adolescents concerned attitudes, behavior and knowledge. HCPs should build trust, with mutual understanding, to prevent adolescents from feeling pressure to provide information about their feelings and problems [30]. Gender preferences varied in the studies. Coates [31] found that some adolescents liked to be allocated to an HCP of the same gender. Some adolescents (four females and one male) in Buston’s study [32] felt uncomfortable talking to male HCPs. Young female HCPs were described as more popular, because of their youthful outlook and spirit [33].

Adolescents described certain qualities in their relationship with HCPs. Having someone to talk to and being heard were important, and such relationships were described as “a good fit*”* [31] and “we just clicked” [32]. Adolescents appreciated HCPs maintaining the relationship despite difficulties. When this occurred, it was perceived as a facilitator to PCC [34]. Hart and colleagues [33] noted that PCC skills seemed to be more relevant to the adolescents than treatment approaches. For example, one adolescent’s response to a question about the impact of the HCP relationship was: “He was a good listener” [33]. Challenges in the therapeutic relationship could have a negative effect on treatment [33–35]. Sometimes such problems increased the adolescent’s hopelessness and distress in treatment [35]. For adolescents with psychosis, experiences of not being heard or respected regarding their personal viewpoints were identified as triggers for incidents involving destructive behaviour [36].

Concerns were raised regarding transparency in the treatment process. Transparency was linked to perceptions of choice and sense of control in treatment [37]. The adolescents recommended that HCPs’ communication should be free of judgment, blame and criticism. Staff turnover was noted as a key issue that negatively impacted the therapeutic alliance [35, 38]. Several adolescents noted how difficult it was to have “opened your heart” to someone who left, and then to feel vulnerable when needing to re-tell their story to a new HCP [32, 35, 38].

### Theme 2: need for active engagement in treatment (*n* = 19)

All studies described how adolescents wanted to be actively engaged in all stages of treatment. Coyne and colleagues [38] underlined the importance of hearing stories directly from adolescents and involving thems in every step of their treatment. According to Munford and Sanders [39], interventions could be unsuccessful if adolescents were unable to connect and engage with HCPs. Buston [32] suggested that development of HCPs’ empathic communication skills was a key factor to improve mental healthcare. Findings by Byczkowski and colleagues [40] suggested prioritizing effective communication in order for HCPs to increase adolescent involvement in their care decisions.

Adolescents expressed preferences for being recognized as experts by experience [31], pointing out that healthcare services need to be responsive to the trend of treating adolescent clients in a more “adult” way [35], or as teenagers in their own right [32]. In several studies, adolescents had clear treatment preferences on how care and treatment could be improved to enhance self-engagement and outcomes [30, 31, 34, 39, 41–43]. Davison and colleagues [34] pointed out the importance of listening genuinely to the views of adolescents and taking them seriously. Being listened to and being encouraged to work with HCPs in decision-making processes were common themes from adolescents about how services could be improved [31, 39].

Personal control over life choices was regarded as important, as adolescents valued having a sense of choice [36]. Some studies also found that adolescents preferred the opportunity to discuss their problems to merely receiving medication [32, 44]. Davison and colleagues [34] indicated that adolescents wanted to give feedback about their experiences in a meaningful way, and that exclusion from decision-making processes increased the likelihood of disengagement from treatment.

Adolescents were sensitive to stigma attached to mental health problems. Studies showed that negative beliefs about mental health services and the stigma attached to these were barriers [34, 38]. Davison and colleagues [34] suggested that modifying access to mental health services by providing information in various settings such as schools and colleges could reduce the stigma surrounding mental health.

### Theme 3: different experiences due to time of data collection (*n* = 19)

The third theme focused on differences in adolescents’ experiences based on the data collection period (see Table 2). In some studies, data were collected at the first meeting [41, 43, 45–47]. Uncertainty was a key concept; adolescents reported not knowing or being unsure of what to expect when they entered treatment. Salamone-Violi and colleagues [47] found that fear of the unknown made adolescents feel uncertain as to whether they would be accepted by other patients and HCPs in an inpatient unit. Midgley and colleagues [45] reported that uncertainty was perhaps the most consistently striking feature of their interviews, as the words “don’t know” or “dunno” were used at least once in 70 of the 77 interviews. Stafford and colleagues [43] found that adolescents were not always directly asked for their understanding of why they were attending treatment. Instead, variations of the question “Why are you here?” were used [43]. The study suggested that asking adolescents directly in the first appointment would improve understanding of how they perceived their problems and engagement in therapy [43]. Ronzoni and Dogra [46] indicated that information needs to be age appropriate and relevant to adolescents.

When interviews were conducted during treatment, adolescents found HCPs’ attitude more positive; the HCPs were more engaged and less cold than they had imagined [48]. Adolescents found treatment more complex than they had anticipated, being surprised by the amount of work and time they needed to devote to their recovery [41, 48]. Grealish and colleagues [36] reported that adolescents felt more empowered when they received detailed information and explanations about their treatment. Conversely, when information was unclear, adolescents experienced frustration and isolation, and felt that they were not receiving help [36]. Studies found that self-engagement through shared experiences with fellow adolescents was perceived as satisfying in inpatient facilities, highlighting the power of peer solidarity [41, 47].

Studies that conducted interviews at the end of or after treatment reported that adolescents felt better able to solve problems and cope with life [41]. However, many reported uncertainty about the future [39, 41]. Adolescents described how their relationships with HCPs were often prematurely ended before they were prepared to leave treatment, recognizing that building relationships with HCPs was a gradual process that involved emotional investment [35]. In the study by Munford and Sanders [39], adolescents felt unsure about who would be caring for them and what was happening.

### Theme 4: treatment context and healthcare system (*n* = 13)

This theme included adolescents’ perceptions of the environment and how it affected their mental health. Physical surroundings played an important role, where more informal and homely settings were favored over sterile hospital environments [30]. Adolescents used words such as “warm, welcoming” [30, 40, 48], “safe”, and “comfortable” to describe the physical environment [30, 41, 47]. There were reports of poor food and hygiene in inpatient settings [32].

Adolescents emphasized that mental health treatment should be more readily available in terms of medical care at short notice, continuity, and quick access [30, 34]. Harper and colleagues [35] highlighted developmentally attuned services, implying that services should be based on addressing individual needs rather than being divided into age groups. In another study where the HCPs sent phone text messages to adolescents to remind them about appointments, the adolescents regarded this information as convenient and helpful [44].

Adolescents stated that they wanted daily structure and activities. They recommended using games or activities initially to get to know the HCP [34]. LeFrançois [49] highlighted the importance of choosing between different activities, depending on interests, individual needs, and mood on a given day.

### Theme 5: adolescent-caregiver interaction (*n* = 10)

The final theme concerns the involvement of caregivers in supporting adolescents in mental health treatment and varying perceptions of this role. Hayes and colleagues [41] indicated that caregivers, who may be biological parents or legal guardians, need to be involved in this way. Confidentiality was important to adolescents, but caregivers did not always perceive the issue of confidentiality in the same way [40]. Harper and colleagues [35] found that adolescents did not always describe caregiver involvement in positive terms, as it was reported as being overly intrusive at times, or setting limits for what the adolescents shared with their HCP [40]. Ronzoni and Dogra [46] suggested a joint formulation of treatment goals between adolescents and their caregivers to enhance collaboration.

Some adolescents realized that it would be hard to engage in services without their caregivers [33, 36]. Adolescents with psychosis underlined that their parents were the ones they trusted the most in taking decisions on their behalf, viewing them as their key resource [36]. Coyne and colleagues [38] found that adolescents described being unwilling or unable to speak openly to an HCP with a parent present, preferring separate consultations.

Adolescents highlighted the importance of providing parents with continued support when needed [32], particularly during periods when the adolescents have experienced poor mental health, as they relied on their parents to make decisions on their behalf [36].

## Discussion

The current scoping review aimed to explore the overall experiences of adolescents’ self-engagement and to identify factors that are important to facilitate self-engagement in mental health treatment. The results were grouped into five themes: the therapeutic alliance, the need for active engagement in treatment, different experiences due to time of data collection, treatment context and healthcare system, and adolescent-caregiver interaction. Our major findings indicate a broadening conceptual understanding of self-engagement, implying that self-engagement is not static, but influenced by all the five themes. Our findings support the review of Hawke and colleagues [4], which stated that mental health services need to integrate adolescents’ voices in terms of self-engagement at all levels of an organization. This is in accordance with the proposal by the WHO [9, 10] that adolescents’ needs, wishes and preferences must be a central factor in informing and designing their treatment [11].

This scoping review of 19 studies demonstrates that there is limited knowledge on the topic of self-engagement in the field of PCC regarding adolescents in mental health treatment, confirming previous findings that adolescents are rarely actively involved in their mental health treatment [15, 20]. Our scoping review contributes to the existing literature by including adolescents’ voices for a wider range of self-engagement in treatment. Thus, the findings imply that self-engagement can be seen as a broad and complex concept, influenced by individual factors, contextual factors and the relationship between adolescents, caregivers and HCPs. This multilevel complexity may partly explain why mental healthcare has been slow to respond to recommendations made by adolescents.

HCPs who elicit adolescents’ perspectives on their mental health symptoms may be more likely to increase adolescents’ self-engagement; it will then develop in dynamic interaction between contextual factors and the person’s unique personality and symptom burden [12, 16]. In this connection, adolescents in the studies consistently mentioned the importance of the therapeutic alliance. This main theme fits remarkably well with the principles of PCC, which emphasize the ability of HCPs to be respectful and supportive, and to listen to adolescents’ needs [1, 2]. This finding also addresses the complexity of the role of caregivers in adolescents’ mental health treatment. This indicates that the principles of PCC need to be applied in a way that meets adolescents’ expectations that adults will listen to them, appreciate them and reflect on their opinions [6]. Adolescents’ concerns about privacy and confidentiality in interaction with caregivers reflect possible conflicts in the adolescent-caregiver relationship. At the same time, caregivers need to be involved to support adolescents in mental health treatment [41]. PCC approaches recognize the importance of both adolescents’ and caregivers’ perspectives but balancing them is challenging [15]. The findings in this study are in line with the review by Lynch and colleagues [5], indicating that HCPs need to prioritize the core components of trust and confidentiality as essential in the provision of meaningful mental health treatment for adolescents.

The time of data collection is one of the five main themes in our review. Data were collected at three distinct time points: before, during, and after treatment. These time points revealed different adolescent experiences related to self-engagement. To ask adolescents at the beginning of treatment about their expectations for the upcoming treatment could lead to less uncertainty and increased understanding of their perspectives on their problems. Information and explanations during treatment, especially at the start and at the end, were of importance. Adolescents who were interviewed at the end of or after treatment reported feeling unsure about their future and had a sense that the treatment was terminated prematurely. In this way, self-engagement can be understood as an ongoing process. This finding is consistent with the review conducted by Dixon and colleagues [16], emphasizing the importance of patients’ sustained self-engagement throughout treatment.

The findings underline the importance of treatment context and daily structure for self-engagement. Adolescents wanted mental health treatment settings to be more relaxed and informal, which is a consistent finding in the literature on mental health services in general [14].

In acknowledging the transferability of findings, it is essential to consider variations in healthcare systems and cultural factors. While self-engagement in mental health treatment is advocated as an adolescent’s right to live a fulfilling adult life [8–10], its realization depends on the complexity of healthcare systems and cultural nuances.

### Clinical relevance and research implications

The results of this review support mental healthcare based on a PCC approach [1, 2], involving values such as acceptance, respect, professionalism, communication, and acceptance [6]. The findings clearly show that adolescents want to be involved in their mental health treatment. Adolescents express a clear interest in providing constructive feedback to improve the quality of their care. Moreover, they emphasize the importance of active participation in decision-making processes. In fact, a need for active self-engagement in treatment was found to be a core value across the five themes. The UN Convention on the Rights of the Child states that adolescents should have the right to express their views and their opinion should be given due weight in all matters affecting them [8].

As findings from the current review show, self-engagement is considered essential for PCC [4]. This review provides insight into the complexity of integrating active self-engagement for adolescents in mental health treatment. HCPs need to be aware that active self-engagement is a broad and complex concept. In order to succeed, HCPs must focus on all the factors involved: individual, contextual, and relational between adolescents, caregivers, and HCPs. Mental health treatment without adolescent self-engagement can lead to worse clinical outcomes and symptom relapse [16]. A clinical contribution from this review is further attention to improvement of HCPs’ communication skills adapted to adolescents’ needs. On a practical level, HCPs should make efforts to listen to adolescents’ own stories and involve them in every step of their treatment [4]. Further, HCPs need to find out what level of caregiver involvement in treatment is desired by the individual adolescent. HCPs also need also to be aware of the legislation that addresses mental healthcare for adolescents and caregivers [6]. This knowledge is crucial to ensure that HCPs navigate legal and ethical considerations effectively when providing care to this population. It will safeguard the rights and well-being of both adolescents and their caregivers.

Our review demonstrates the limited knowledge of PCC in mental health treatment for adolescents. Hence, we suggest further research on adolescents’ voices regarding the broad and complex concept of self-engagement. Moreover, further knowledge is needed to explore the interaction between individual, contextual and relational factors. Cultural background awareness and provision of culturally competent care may be one way to enhance self-engagement [13, 16]. One person-centered tool is the Cultural Formulation Interview (CFI) published in the Diagnostic and Statistical Manual of Mental Disorders, Fifth Edition (DSM-5) [50]. Research shows that use of the CFI is an important step toward PCC [51], as it improves the therapeutic alliance and contributes to subjective exploration of the patient’s health narrative [52, 53].

Further research on specific PCC interventions such as the CFI may strengthen understanding of and evidence for active self-engagement by adolescents in mental health treatment. Due to the limited research on adolescents’ self-engagement, there is a need for a diversity of methods in different clinical contexts. Important here are longitudinal research throughout the treatment process and targeted research on confidentiality to enhance adolescent-caregiver interaction. In addition, further research should explore HCPs’ and caregivers’ voices on self-engagement in mental healthcare to complement these results.

The advent of new technology means that self-engagement can be further improved through the use of digital tools [17]. In our review, adolescents identified benefits of e.g., text messaging to access help on their own [44], indicating that digital tools to increase information and access to support in mental healthcare are an area for future research.

### Limitations

This review was conducted using rigorous and systematic methodology. Nevertheless, the review has some limitations. The literature in the area of PCC in adolescent mental health treatment proved to be limited, which necessitated a broad search, including gray literature. While focusing on breadth over depth, this review may constitute a basis for future systematic reviews to explore in depth specific topics identified here. However, an initial tentative research question would have been of value for a more clearly defined search and for clarity in the inclusion criteria. While broad techniques were employed to identify relevant studies, using text-based search strategies in combination with manual backward citation searching and co-citation searching, we acknowledge that we could have included forward citation searches. This could have yielded recently published material not captured by the text-based search. However, searching for gray literature and the co-citation searches should to some extent compensate in covering recent publications.

Including only qualitative studies in this review may have weakened its scope by overlooking quantitative trends and patterns that provide a more comprehensive understanding of this field. Including both qualitative and quantitative studies could have resulted in more balanced and thorough insight. However, in such an under-researched area a focus on qualitative studies was deemed essential, in terms of the contributions of qualitative methodologies and the need for a more in-depth analysis.

The clinical contexts in this review were dominated by outpatient clinics. Only three studies recruited adolescents from inpatient units. The majority of the studies originated from the United Kingdom, particularly England, followed by Australia. There was variation in the timing of data collection. Only one study was longitudinal, with interviews at three different points during treatment. In some studies, caregivers were part of the data collection, but their level of participation differed. This varying role might have affected how adolescents responded, but it is difficult to confirm this. In addition, the included adolescents had a wide age range and a variety of mental illnesses. Despite this, adolescents’ preferences for self-engagement in mental health treatment seemed to demonstrate more similarities than differences. This should enhance transferability of the findings to a variety of mental healthcare contexts for adolescents.

Although several studies in the identification and selection process reported on adolescents’ perspectives, it is important to note that the age limitation (12–18 years) may have resulted in the exclusion of studies focusing on children or young adults that could be of relevance to adolescents. However, some studies included adolescents under 12 years or above 18 years. We chose to include these studies since most of the participants were in the age range 12–18 years. Additionally, any attempt to standardize methods of data collection in this group may encounter difficulties because of the wide age range of the adolescents. In Ronzoni and Dogra [46], the age range of the adolescents was 6–18 years. A method suitable for a six-year-old may not be appropriate for an 18-year-old, yet the same method is used for a wide age range in many studies. Only five studies used a combination of methods to ascertain adolescents’ views. Worrall-Davies and Marino‐Francis [18] highlighted the importance of using more than one method when eliciting adolescents’ views of mental health treatment, and of ensuring that the research methodology is appropriate to the particular developmental level of the adolescent. In this review, it is possible that the adolescents included were those who had a particularly high level of engagement in mental health treatment. Presumably, adolescents who drop out of mental health treatment were not included in the studies as they are under-represented in the literature [3]. Future research may consider splitting the age range between younger and older adolescents given developmental variations. This approach could provide a more comprehensive understanding of self-engagement, acknowledging potential differences in how younger and older adolescents are able to articulate their views and opinions.

Finally, it is important to acknowledge that the systematic search used to identify studies for inclusion in this review was conducted in January 2022, implying that relevant studies published later were not included.

## Conclusion

The UN [8] and the WHO [9, 10] have called for adolescents’ needs, wishes and preferences to be a vital factor in their mental health treatment. A central focus on a person’s values in treatment is essential to a PCC approach to practice [1, 2]. This review has explored self-engagement, informed by adolescents’ own voices, in mental health treatment. The findings indicate the need for a multilevel and comprehensive understanding of self-engagement that includes individual, contextual, and relational factors. The most important findings were the significance of building a strong therapeutic alliance between the adolescent and the HCP, and adolescents’ attitude of wanting to be actively engaged in their treatment. Results from this review show that active self-engagement is not static, but influenced by all the five main themes. This insight highlights the complexity of facilitating self-engagement for this population. To enhance the practice of PCC in adolescent mental health treatment, HCPs must acknowledge this complexity. Neglecting adolescents’ active self-engagement in their treatment can worsen clinical outcomes. To ensure successful treatment, HCPs should pay further attention to developing communication skills adapted to adolescents’ needs. HCPs need to involve adolescents throughout treatment and consider their preferences for caregiver involvement. Research on adolescents’ voices on self-engagement has been scarce and this study is seen as a key contribution. Further research on specific PCC interventions may strengthen the understanding of and evidence for active self-engagement by adolescents in mental healthcare. Given the limited existing research on adolescents’ self-engagement, it is imperative to employ various research methods across diverse clinical settings. Furthermore, future research should aim to explore the perspectives of HCPs and caregivers on adolescents’ self-engagement in mental health treatment to complement the findings of this review.

## Electronic supplementary material

Below is the link to the electronic supplementary material.

Supplementary Material 1

Supplementary Material 2

## Data Availability

All relevant data are in the manuscript and its supporting information files.
