# Supplementary material for: Adolescents’ voices on self-engagement in mental health treatment: a scoping review
Source: Eur Child Adolesc Psychiatry. 2024 Mar 27;33(12):4083–95. doi: 10.1007/s00787-024-02425-7 (PMC11618195; doi:10.1007/s00787-024-02425-7)
Supplement: Supplementary file 1 — Supplementary Material 1 [file 787_2024_2425_MOESM1_ESM.pdf]

Supplementary Material 1

Search strategy for databases, gray literature and citation search

Adolescents' voices on self-engagement in mental health treatment: a scoping review

European Child & Adolescent Psychiatry

Nina Therese Øversveen Svamo, Research Center for Existential Health, Innlandet Hospital Trust, Norway; Inland Norway University of Applied Sciences Lillehammer, Nina.Therese.Oversveen.Svamo@sykehuset-innlandet.no

Sigrid Helene Kjørven Haug

Valerie DeMarinis

Urd Hertzberg

International and Scandinavian research databases

Search #	Database	Interface	Retrieval date (final search)	Results before duplicate removal
#1	Ovid MEDLINE(R) and Epub Ahead of Print, In-Process, In-Data-Review & Other Non-Indexed Citations and Daily <1946 to February 18, 2022>	Ovid	February 21, 2022	3260
#2	APA PsycInfo <1806 to February Week 2 2022>	Ovid	February 21, 2022	851
#3	Embase <1974 to 2022 February 18>, Medline journals excluded	Ovid	February 21, 2022	191
#4	Cinahl Complete	EbscoHost	February 21, 2022	340
#5	Oria (cut-off after 300 first results)	Primo	November 11, 2021	300
#6	Idunn, Universitetsforlaget (manual selection)	Idunn	November 11, 2021	6
#7	Norart, Nasjonalbiblioteket	Norart.nb.no	November 12, 2021	65
#8	Svemed+ (not updated after January 2020)		November 12, 2021	184
#9	Publicera.se		November 12, 2021	0
#10	Sweplus		November 12, 2021	1
#11	Tidsskrift.dk (manual selection)		November 12, 2021	13

Gray literature registers

Gray literature Registers searched	Retrieval date (final search)
Embase (Ovid)	November 23, 2021
NIH RePORTER	November 23, 2021
ClinicalTrials.gov	November 23, 2021
World Health Organization (WHO) International Clinical Trials Registry Platform (ICTRP),	November 23, 2021
OAIster	November 23, 2021
NARCIS – National Academic Research and Collaborations	November 23, 2021

Supplementary Material 1

Information System (Netherlands)	
Open Grey	November 23, 2021
RIAN (Pathways to Irish Research)	November 23, 2021

Supplementary Material 1

Seed articles used in Citation Gecko and Co-Cites

Citation Gecko and Co-Cites were used for identifying articles cited or co-cited by the seed articles listed below.

Armstrong S, Wammes M, Arcaro J, Hostland A, Summerhurst C, Osuch E (2019) Expectations vs reality: the expectations and experiences of psychiatric treatment reported by young adults at a mood and anxiety outpatient mental health program. Early Interv Psychiatry 13:633-638. https://doi.org/10.1111/eip.12550

Buston K (2002) Adolescents with mental health problems: what do they say about health services? J Adolesc 25:231-242. https://doi.org/10.1006/jado.2002.0463

Byczkowski TL, Kollar LM, Britto MT (2010) Family experiences with outpatient care: do adolescents and parents have the same perceptions? J Adolesc Health 47:92-98. https://doi.org/10.1016/j.jadohealth.2009.12.005 Cairns, A., Dark, F., Kavanagh, D., & McPhail, S. (2015). Exploring functional concerns in help-seeking youth: a qualitative study. Early Interv Psychiatry, 9(3), 228-233. https://doi.org/10.1111/eip.12101

Coates D (2016) Client and parent feedback on a Youth Mental Health Service: the importance of family inclusive practice and working with client preferences. Int J Ment Health Nurs 25:526-535. 10.1111/inm.12240 https://doi.org/10.1111/inm.12240

Coyne I, McNamara N, Healy M, Gower C, Sarkar M, McNicholas F (2015) Adolescents' and parents' views of Child and Adolescent Mental Health Services (CAMHS) in Ireland. J Psychiatr Ment Health Nurs 22:561-569. https://doi.org/10.1111/jpm.12215

Davison J, Zamperoni V, Stain HJ (2017) Vulnerable young people’s experiences of child and adolescent mental health services. Ment Health Rev J 22:95-110. https://doi.org/10.1108/mhrj-09-2016-0016

Draucker, C. B (2005) Processes of mental health service use by adolescents with depression. J Nurs Scholarsh, 37(2), 155-162. https://doi.org/10.1111/j.1547-5069.2005.00028.x

Edwards M, Lawson C, Rahman S, Conley K, Phillips H, Uings R (2016) What does quality healthcare look like to adolescents and young adults? Ask the experts! Clin Med (Northfield Il) 16:146. https://doi.org/10.7861/clinmedicine.16-2-146

Grealish A, Tai S, Hunter A, Morrison AP (2013) Qualitative exploration of empowerment from the perspective of young people with psychosis. Clin Psychol Psychother 20:136-148. https://doi.org/10.1002/cpp.785

Green CA, Wisdom JP, Wolfe L, Firemark A (2012) Engaging youths with serious mental illnesses in treatment: STARS study consumer recommendations. Psychiatr Rehabil J 35:360. https://doi.org/10.1037/h0094494

Harper B, Dickson JM, Bramwell R (2014) Experiences of young people in a 16–18 Mental Health Service. Child Adolesc Ment Health 19:90-96. https://doi.org/10.1111/camh.12024

Hart A, Saunders A, Thomas H (2005) Attuned practice: a service user study of specialist child and adolescent mental health, UK. Epidemiol Psichiatr Soc 14:22-31. https://doi.org/10.1017/s1121189x00001895

McCann TV, Lubman DI (2012) Young people with depression and their satisfaction with the quality of care they receive from a primary care youth mental health service: a qualitative study. J Clin Nurs 21:2179-2187. https://doi.org/10.1111/j.1365-2702.2012.04086.x

Munford R, Sanders J (2016) Understanding service engagement: young people’s experience of service use. J Soc Work 16:283-302. https://doi.org/10.1177/1468017315569676

Persson S, Hagquist C, Michelson D (2017) Young voices in mental health care: exploring children’s and adolescents’ service experiences and preferences. Clin Child Psychol Psychiatry 22:140-151. https://doi.org/10.1177/1359104516656722

Ronzoni P, Dogra N (2012) Children, adolescents and their carers’ expectations of child and adolescent mental health services (CAMHS). Int J Soc Psychiatry 58:328-336. https://doi.org/10.1177/0020764010397093

Salamone-Violi GM, Chur-Hansen A, Winefield HR (2015) ‘I don't want to be here but I feel safe’: Referral and admission to a child and adolescent psychiatric inpatient unit: the young person's perspective. Int J Ment Health Nurs 24:569-576. https://doi.org/10.1111/inm.12163

Stafford V, Hutchby I, Karim K, O’Reilly M (2016) “Why are you here?” Seeking children’s accounts of their presentation to Child and Adolescent Mental Health Service (CAMHS). Clin Child Psychol Psychiatry 21:3-18. https://doi.org/10.1177/1359104514543957

Supplementary Material 1

Complete search strategies for database searches #1 through #11

Search #1

Ovid MEDLINE(R) and Epub Ahead of Print, In-Process, In-Data-Review & Other Non-Indexed Citations and Daily <1946 to February 18, 2022>

1	Patient-Centered Care.mp. or exp Patient-Centered Care/	26730
2	Physician-Patient Relations.mp. or exp Physician-Patient Relations/	75587
3	Patient Satisfaction.mp. or exp Patient Satisfaction/	118203
4	Patient Preference.mp. or exp Patient Preference/	14387
5	exp Decision Making, Shared/	1449
6	Patient Participation.mp. or exp Patient Participation/	30119
7	Professional-Patient Relations.mp. or exp Professional-Patient Relations/	147056
8	Nurse-Patient Relations.mp. or exp Nurse-Patient Relations/	36005
9	Doctor-Patient Relationship.mp.	3764
10	Shared Decision Making.mp.	11389
11	Doctor Patient Relation.mp.	71
12	Personalized Medicine.mp.	15491
13	Nurse Patient Relationship.mp.	908
14	Patient Centered Care.mp.	25694
15	Therapeutic Processes.mp.	500
16	Client Satisfaction.mp.	1283
17	Client Participation.mp.	127
18	Client Centered Therapy.mp.	87
19	Patient-Centredness.mp.	530
20	Patient-Centeredness.mp.	1258
21	Patient-Centered communication.mp.	687
22	communication patient-doctor.mp.	3
23	communication patient-nurse.mp.	1
24	communication patient-clinician.mp.	3
25	patient-physician decision making.mp.	32
26	physician decision making.mp.	468
27	patient communication behavior.mp.	7
28	individualized care.mp.	1454
29	personalized care.mp.	1203
30	person-centered care.mp.	1410
31	PCC.mp.	11561
32	Client centered care.mp.	132
33	resident centered care.mp.	57
34	personal customization.mp.	5
35	Therapeutic Relationship.mp.	2847
36	user participation.mp.	318
37	holistic care.mp.	2265
38	Psychologist-Patient Relations.mp.	1
39	Psychologist-Patient relationship.mp.	0
40	Psychologist Patient relationship.mp.	0
41	Psychologist Patient relations.mp.	1
42	patient-psychologist decision making.mp.	0
43	Patient Acceptance of Health Care.mp. or "Patient Acceptance of Health Care"/	52820
44	(patient engagement or psychological engagement).mp.	4019
45	Cooperative Behavior/ or cooperative behavior.mp.	46174

Supplementary Material 1

46 patient involvement.mp. 3126

47 1 or 2 or 3 or 4 or 5 or 6 or 7 or 8 or 9 or 10 or 11 or 12 or 13 or 14 or 15 or 16 or 17 or 18 or 19 or 20 or 21 or 22 or 23 or 24 or 25 or 26 or 27 or 28 or 29 or 30 or 31 or 32 or 33 or 34 or 35 or 36 or 37 or 38 or 39 or 40 or 41 or 42 or 43 or 44 or 45 or 46 432838

48 Child Psychiatry.mp. or exp Child Psychiatry/ 7228

49 Psychology, Child.mp. or exp Psychology, Child/ 13547

50 Adolescent Psychiatry.mp. or exp Adolescent Psychiatry/ 4860

51 Psychology, Adolescent.mp. or exp Psychology, Adolescent/ 13796

52 Child.mp. or exp Child/ 2257416

53 Child Health.mp. or exp Child Health/ 50449

54 exp Adolescent/ or Adolescent.mp. 2195954

55 Adolescent Health.mp. or exp Adolescent Health/ 12862

56 Young Adult.mp. or exp Young Adult/ 1003781

57 52 or 53 or 54 or 55 or 56 3887197

58 Psychiatry.mp. or exp Psychiatry/ 144818

59 Psychology.mp. or exp Psychology/ 1290814

60 58 or 59 1400173

61 57 and 60 472526

62 Adolescent Health Services.mp. or exp Adolescent Health Services/ 5933

63 Child Health Services.mp. or exp Child Health Services/ 27702

64 Mental Health Services.mp. or exp Mental Health Services/ 111766

65 limit 64 to ("preschool child (2 to 5 years)" or "child (6 to 12 years)" or "adolescent (13 to 18 years)" or "young adult (19 to 24 years)") 29871

66 62 or 63 or 65 59663

67 48 or 49 or 50 or 51 or 61 472526

68 66 and 67 17695

69 47 and 68 3260

Search #2

APA PsycInfo <1806 to February Week 2 2022>

1 exp Patient Centered Care/ or Patient Centered Care.mp. 4860

2 Therapeutic Processes.mp. or exp Therapeutic Processes/ 80451

3 Client Satisfaction.mp. or exp Client Satisfaction/ 7837

4 Client Participation.mp. or exp Client Participation/ 2897

5 Decision Making.mp. or exp Decision Making/ 183559

6 Client Centered Therapy.mp. or exp Client Centered Therapy/ 3753

7 patient-centred care.mp. 730

8 physician-patient relations.mp. 13119

9 patient satisfaction.mp. 14167

10 patient preference.mp. 2361

11 shared decision making.mp. 3325

12 patient participation.mp. 6077

13 professional-patient relationship.mp. 63

14 nurse-patient relations.mp. 4578

15 doctor-patient relationship.mp. 2000

16 doctor patient relation.mp. 36

17 personali?ed medicine.mp. 1088

Supplementary Material 1

18	nurse patient relationship.mp.	448
19	patient-cent?redness.mp.	664
20	patient-cent?red communication.mp.	464
21	communication patient-doctor.mp.	0
22	communication patient-nurse.mp.	0
23	communication patient-clinician.mp.	2
24	patient-physician decision making.mp.	8
25	physician decision making.mp.	102
26	patient communication behavior.mp.	6
27	individuali?ed care.mp.	593
28	personali?ed care.mp.	307
29	person-cent?red care.mp.	1535
30	client cent?red care.mp.	168
31	PCC.mp.	1557
32	resident cent?red care.mp.	42
33	personal customi?ation.mp.	1
34	therapeutic relationship.mp.	8687
35	user participation.mp.	373
36	(Holistic care or Psychologist-Patient Relations or Psychologist-Patient relationship or Psychologist Patient relationship or Psychologist Patient relations or patient-psychologist decision making).mp.	773
37	exp Participation/ or participation.mp.	124026
38	involvement.mp. or exp Involvement/	123825
39	exp Psychological Engagement/ or Psychological engagement.mp.	12695
40	patient engagement.mp.	1149
41	Cooperative Behavior.mp.	10275
42	cooperation/ or cooperation.mp.	35846
43	engagement.mp.	84660
44	patient involvement.mp.	1106
45	Patient Acceptance of Health Care.mp.	12215
46	1 or 2 or 3 or 4 or 5 or 6 or 7 or 8 or 9 or 10 or 11 or 12 or 13 or 14 or 15 or 16 or 17 or 18 or 19 or 20 or 21 or 22 or 23 or 24 or 25 or 26 or 27 or 28 or 29 or 30 or 31 or 32 or 33 or 34 or 35 or 36 or 37 or 38 or 39 or 40 or 41 or 42 or 43 or 44 or 45	617018
47	Child Psychiatry.mp. or exp Child Psychiatry/	9837
48	Child Psychology.mp. or exp Child Psychology/	6195
49	Adolescent Psychiatry.mp. or exp Adolescent Psychiatry/	8287
50	Adolescent Psychology.mp. or exp Adolescent Psychology/	5218
51	Adolescent Health.mp. or exp Adolescent Health/	7724
52	child.mp.	500169
53	Adolescent.mp.	407457
54	52 or 53	766868
55	exp Psychiatry/ or Psychiatry.mp.	106994
56	Psychology.mp. or exp Psychology/	536442
57	55 or 56	623994
58	54 and 57	106305
59	47 or 48 or 49 or 50 or 51 or 58	113111
60	Mental Health Services.mp. or exp Mental Health Services/	70043
61	59 and 60	4204
62	46 and 61	851

Supplementary Material 1
Search #3

Embase <1974 to 2022 February 18>

1 patient care/ or advance care planning/ or case finding/ or case management/ or clinical handover/ or collaborative care team/ or cultural safety/ or holistic care/ or night care/ or patient assessment/ or patient care planning/ or patient comfort/ or patient decision making/ or patient isolation/ or patient lifting/ or patient monitoring/ or patient positioning/ or patient referral/ or patient scheduling/ or patient selection/ or peroperative care/ or postanesthesia care/ or rapid response team/ or retention in care/ or shared medical appointment/ or subacute care/ 761967
2 doctor patient relation.mp. or exp doctor patient relationship/ 116748
3 Doctor-Patient Relationship.mp. or exp doctor patient relation/ 11342
4 patient satisfaction.mp. or exp patient satisfaction/ 164595
5 shared decision making.mp. or exp shared decision making/ 19204
6 patient participation.mp. or exp patient participation/ 33213
7 personalized medicine.mp. or exp personalized medicine/ 65748
8 nurse patient relationship.mp. or exp nurse patient relationship/ 33393
9 professional-patient relationship.mp. or exp professional-patient relationship/ 51935
10 patient cent?red care.mp. 12118
11 physician-patient relations.mp. 1346
12 patient preference.mp. or exp patient preference/ 26417
13 decision making.mp. or exp decision making/ 493791
14 therapeutic processes.mp. 731
15 client satisfaction.mp. 1704
16 client participation.mp. 179
17 client cent?red therapy.mp. 438
18 nurse-patient relations.mp. or nurse patient relationship/ 33247
19 patient-cent?redness.mp. 2170
20 patient-cent?red communication.mp. 1125
21 communication patient-doctor.mp. 4
22 communication patient-nurse.mp. 0
23 communication patient-clinician.mp. 2
24 Patient-Physician Decision Making.mp. 47
25 Physician Decision Making.mp. 703
26 patient communication behavior.mp. 7
27 individuali?ed care.mp. 2660
28 personali?ed care.mp. 2295
29 person-cent?red care.mp. 3583
30 PCC.mp. 15388
31 client cent?red care.mp. 265
32 resident cent?red care.mp. 79
33 personal customi?ation.mp. 3
34 therapeutic relationship.mp. 4223
35 user participation.mp. 379
36 (Holistic care or Psychologist-Patient Relations or Psychologist-Patient relationship or Psychologist Patient relationship or Psychologist Patient relations or patient-psychologist decision making).mp. 5947
37 participation.mp. 245950
38 involvement.mp. 667240
39 Psychological engagement.mp. 41
40 patient engagement.mp. or patient engagement/ 5917
41 Cooperative Behavior.mp. or cooperation/ 45754

Supplementary Material 1

42 cooperation.mp. 162714
43 engagement.mp. 111591
44 patient involvement.mp. 4281
45 Patient Acceptance of Health Care.mp. or patient attitude/ 73347
46 patient attitude.mp. 73270
47 1 or 2 or 3 or 4 or 5 or 6 or 7 or 8 or 9 or 10 or 11 or 12 or 13 or 14 or 15 or 16 or 17 or 18 or 19 or 20 or 21 or 22 or 23 or 24 or 25 or 26 or 27 or 28 or 29 or 30 or 31 or 32 or 33 or 34 or 35 or 36 or 37 or 38 or 39 or 40 or 41 or 42 or 43 or 44 or 45 or 46 2624675
48 Child Psychiatry.mp. or exp child psychiatry/ 22751
49 Child Psychology.mp. or exp child psychology/ 16669
50 exp child/ or Child.mp. 3083227
51 Child Health.mp. or exp child health/ 87784
52 Child Health Care.mp. or exp child health care/ 99290
53 exp adolescent/ or Adolescent.mp. 1694043
54 Adolescent Health.mp. or exp adolescent health/ 15737
55 Young Adult.mp. or exp young adult/ 475610
56 50 or 51 or 52 or 53 or 54 or 55 4168195
57 exp psychiatry/ or Psychiatry.mp. 182975
58 Psychology.mp. or exp psychology/ 431544
59 57 or 58 598824
60 56 and 59 179759
61 48 or 49 or 60 179759
62 exp mental health service/ or Mental Health Service*.mp. 68850
63 47 and 61 and 62 1709
64 limit 63 to exclude medline journals 191

Search #4

Database - CINAHL Complete

Interface - EBSCOhost Research Databases

Search Screen - Advanced Search

Monday, February 21, 2022 12:40:15 PM

**#**	**Query**	**Limiters/Expanders**	**Last Run Via**	**Results**
S74	S51 AND S73	Expanders - Apply equivalent subjects	Interface - EBSCOhost Research Databases	340
		Search modes - Boolean/Phrase	Search Screen - Advanced Search	
			Database - CINAHL Complete	
S73	S68 AND S72	Expanders - Apply equivalent subjects	Interface - EBSCOhost Research Databases	2,24

Supplementary Material 1

		Search modes - Boolean/Phrase	Search Screen - Advanced Search	
			Database - CINAHL Complete	
S72	S69 OR S70 OR S71	Expanders - Apply equivalent subjects	Interface - EBSCOhost Research Databases	109,142
		Search modes - Boolean/Phrase	Search Screen - Advanced Search	
			Database - CINAHL Complete	
S71	""Child Health Services""	Expanders - Apply equivalent subjects	Interface - EBSCOhost Research Databases	8,929
		Search modes - Boolean/Phrase	Search Screen - Advanced Search	
			Database - CINAHL Complete	
S70	(MH "Adolescent Health Services") OR ""Adolescent Health Services""	Expanders - Apply equivalent subjects	Interface - EBSCOhost Research Databases	2,999
		Search modes - Boolean/Phrase	Search Screen - Advanced Search	
			Database - CINAHL Complete	
S69	(MH "Mental Health Services+") OR ""Mental Health Services""	Expanders - Apply equivalent subjects	Interface - EBSCOhost Research Databases	98,94
		Search modes - Boolean/Phrase	Search Screen - Advanced Search	
			Database - CINAHL Complete	
S68	S63 OR S64 OR S65 OR S66 OR S67	Expanders - Apply equivalent subjects	Interface - EBSCOhost Research Databases	44,811

Supplementary Material 1

		Search modes - Boolean/Phrase	Search Screen - Advanced Search	
			Database - CINAHL Complete	
S67	S59 AND S62	Expanders - Apply equivalent subjects	Interface - EBSCOhost Research Databases	30,804
		Search modes - Boolean/Phrase	Search Screen - Advanced Search	
			Database - CINAHL Complete	
S66	(MH "Adolescent Psychology") OR "Adolescent Psychology"	Expanders - Apply equivalent subjects	Interface - EBSCOhost Research Databases	3,929
		Search modes - Boolean/Phrase	Search Screen - Advanced Search	
			Database - CINAHL Complete	
S65	""Adolescent Psychiatry""	Expanders - Apply equivalent subjects	Interface - EBSCOhost Research Databases	2,914
		Search modes - Boolean/Phrase	Search Screen - Advanced Search	
			Database - CINAHL Complete	
S64	(MH "Child Psychology") OR "Child Psychology"	Expanders - Apply equivalent subjects	Interface - EBSCOhost Research Databases	17,919
		Search modes - Boolean/Phrase	Search Screen - Advanced Search	
			Database - CINAHL Complete	
S63	(MH "Child Psychiatry") OR "Child Psychiatry"	Expanders - Apply equivalent subjects	Interface - EBSCOhost Research Databases	2,657

Supplementary Material 1

		Search modes - Boolean/Phrase	Search Screen - Advanced Search	
			Database - CINAHL Complete	
S62	S60 OR S61	Expanders - Apply equivalent subjects	Interface - EBSCOhost Research Databases	102,849
		Search modes - Boolean/Phrase	Search Screen - Advanced Search	
			Database - CINAHL Complete	
S61	(MH "Psychology+") OR "Psychology	Expanders - Apply equivalent subjects	Interface - EBSCOhost Research Databases	71,792
		Search modes - Boolean/Phrase	Search Screen - Advanced Search	
			Database - CINAHL Complete	
S60	(MH "Psychiatry+") OR "Psychiatry"	Expanders - Apply equivalent subjects	Interface - EBSCOhost Research Databases	33,544
		Search modes - Boolean/Phrase	Search Screen - Advanced Search	
			Database - CINAHL Complete	
S59	S52 OR S53 OR S54 OR S55 OR S56 OR S57 OR S58	Expanders - Apply equivalent subjects	Interface - EBSCOhost Research Databases	1,366,570
		Search modes - Boolean/Phrase	Search Screen - Advanced Search	
			Database - CINAHL Complete	
S58	(MH "Young Adult") OR "Young Adult"	Expanders - Apply equivalent subjects	Interface - EBSCOhost Research Databases	281,952

Supplementary Material 1

		Search modes - Boolean/Phrase	Search Screen - Advanced Search	
			Database - CINAHL Complete	
S57	(MH "Adolescent Health") OR "Adolescent Health"	Expanders - Apply equivalent subjects	Interface - EBSCOhost Research Databases	13,841
		Search modes - Boolean/Phrase	Search Screen - Advanced Search	
			Database - CINAHL Complete	
S56	""children""	Expanders - Apply equivalent subjects	Interface - EBSCOhost Research Databases	795,232
		Search modes - Boolean/Phrase	Search Screen - Advanced Search	
			Database - CINAHL Complete	
S55	""Adolescent""	Expanders - Apply equivalent subjects	Interface - EBSCOhost Research Databases	157,722
		Search modes - Boolean/Phrase	Search Screen - Advanced Search	
			Database - CINAHL Complete	
S54	(MH "Adolescence+") OR "Adolescence"	Expanders - Apply equivalent subjects	Interface - EBSCOhost Research Databases	581,639
		Search modes - Boolean/Phrase	Search Screen - Advanced Search	
			Database - CINAHL Complete	
S53	(MH "Child Health") OR "Child Health"	Expanders - Apply equivalent subjects	Interface - EBSCOhost Research Databases	37,834

Supplementary Material 1

		Search modes - Boolean/Phrase	Search Screen - Advanced Search	
			Database - CINAHL Complete	
S52	(MH "Child+") OR "Child"	Expanders - Apply equivalent subjects	Interface - EBSCOhost Research Databases	823,276
		Search modes - Boolean/Phrase	Search Screen - Advanced Search	
			Database - CINAHL Complete	
S51	S1 OR S2 OR S3 OR S4 OR S5 OR S6 OR S7 OR S8 OR S9 OR S10 OR S11 OR S12 OR S13 OR S14 OR S15 OR S16 OR S17 OR S18 OR S19 OR S20 OR S21 OR S22 OR S23 OR S24 OR S25 OR S26 OR S27 OR S28 OR S29 OR S30 OR S31 OR S32 OR S33 OR S34 OR S35 OR S36 OR S37 OR S38 OR S39 OR S40 OR S41 OR S42 OR S43 OR S44 OR S45 OR S46 OR S47 OR S48 OR S49 OR S50	Expanders - Apply equivalent subjects	Interface - EBSCOhost Research Databases	581,033
		Search modes - Boolean/Phrase	Search Screen - Advanced Search	
			Database - CINAHL Complete	
S50	"Patient Acceptance of Health Care"	Expanders - Apply equivalent subjects	Interface - EBSCOhost Research Databases	1

Supplementary Material 1

		Search modes - Boolean/Phrase	Search Screen - Advanced Search	
			Database - CINAHL Complete	
S49	"patient involvement"	Expanders - Apply equivalent subjects	Interface - EBSCOhost Research Databases	1,731
		Search modes - Boolean/Phrase	Search Screen - Advanced Search	
			Database - CINAHL Complete	
S48	"engagement"	Expanders - Apply equivalent subjects	Interface - EBSCOhost Research Databases	45,339
		Search modes - Boolean/Phrase	Search Screen - Advanced Search	
			Database - CINAHL Complete	
S47	"cooperation"	Expanders - Apply equivalent subjects	Interface - EBSCOhost Research Databases	10,431
		Search modes - Boolean/Phrase	Search Screen - Advanced Search	
			Database - CINAHL Complete	
S46	(MH "Cooperative Behavior") OR "Cooperative Behavior"	Expanders - Apply equivalent subjects	Interface - EBSCOhost Research Databases	8,936
		Search modes - Boolean/Phrase	Search Screen - Advanced Search	
			Database - CINAHL Complete	
S45	"patient engagement"	Expanders - Apply equivalent subjects	Interface - EBSCOhost Research Databases	2,373

Supplementary Material 1

		Search modes - Boolean/Phrase	Search Screen - Advanced Search	
			Database - CINAHL Complete	
S44	"Psychological engagement"	Expanders - Apply equivalent subjects	Interface - EBSCOhost Research Databases	24
		Search modes - Boolean/Phrase	Search Screen - Advanced Search	
			Database - CINAHL Complete	
S43	"involvement"	Expanders - Apply equivalent subjects	Interface - EBSCOhost Research Databases	86,144
		Search modes - Boolean/Phrase	Search Screen - Advanced Search	
			Database - CINAHL Complete	
S42	"participation"	Expanders - Apply equivalent subjects	Interface - EBSCOhost Research Databases	112,317
		Search modes - Boolean/Phrase	Search Screen - Advanced Search	
			Database - CINAHL Complete	
S41	""patient-psychologist decision making""	Expanders - Apply equivalent subjects	Interface - EBSCOhost Research Databases	0
		Search modes - Boolean/Phrase	Search Screen - Advanced Search	
			Database - CINAHL Complete	
S40	""psychologist-patient relations*""	Expanders - Apply equivalent subjects	Interface - EBSCOhost Research Databases	1

Supplementary Material 1

		Search modes - Boolean/Phrase	Search Screen - Advanced Search	
			Database - CINAHL Complete	
S39	(MH "Holistic Care") OR "Holistic Care"	Expanders - Apply equivalent subjects	Interface - EBSCOhost Research Databases	7,191
		Search modes - Boolean/Phrase	Search Screen - Advanced Search	
			Database - CINAHL Complete	
S38	""user participation""	Expanders - Apply equivalent subjects	Interface - EBSCOhost Research Databases	296
		Search modes - Boolean/Phrase	Search Screen - Advanced Search	
			Database - CINAHL Complete	
S37	""therapeutic relationship""	Expanders - Apply equivalent subjects	Interface - EBSCOhost Research Databases	16,928
		Search modes - Boolean/Phrase	Search Screen - Advanced Search	
			Database - CINAHL Complete	
S36	""personal customi?ation""	Expanders - Apply equivalent subjects	Interface - EBSCOhost Research Databases	1
		Search modes - Boolean/Phrase	Search Screen - Advanced Search	
			Database - CINAHL Complete	
S35	""PCC""	Expanders - Apply equivalent subjects	Interface - EBSCOhost Research Databases	3,011

Supplementary Material 1

		Search modes - Boolean/Phrase	Search Screen - Advanced Search	
			Database - CINAHL Complete	
S34	""resident-centred care""	Expanders - Apply equivalent subjects	Interface - EBSCOhost Research Databases	13
		Search modes - Boolean/Phrase	Search Screen - Advanced Search	
			Database - CINAHL Complete	
S33	""resident-centered care""	Expanders - Apply equivalent subjects	Interface - EBSCOhost Research Databases	49
		Search modes - Boolean/Phrase	Search Screen - Advanced Search	
			Database - CINAHL Complete	
S32	""client-centred care""	Expanders - Apply equivalent subjects	Interface - EBSCOhost Research Databases	104
		Search modes - Boolean/Phrase	Search Screen - Advanced Search	
			Database - CINAHL Complete	
S31	""client-centered care""	Expanders - Apply equivalent subjects	Interface - EBSCOhost Research Databases	16,228
		Search modes - Boolean/Phrase	Search Screen - Advanced Search	
			Database - CINAHL Complete	
S30	""person-centred care""	Expanders - Apply equivalent subjects	Interface - EBSCOhost Research Databases	1,576

Supplementary Material 1

		Search modes - Boolean/Phrase	Search Screen - Advanced Search	
			Database - CINAHL Complete	
S29	""person-centered care""	Expanders - Apply equivalent subjects	Interface - EBSCOhost Research Databases	16,722
		Search modes - Boolean/Phrase	Search Screen - Advanced Search	
			Database - CINAHL Complete	
S28	""personali?ed care""	Expanders - Apply equivalent subjects	Interface - EBSCOhost Research Databases	859
		Search modes - Boolean/Phrase	Search Screen - Advanced Search	
			Database - CINAHL Complete	
S27	""individualized care""	Expanders - Apply equivalent subjects	Interface - EBSCOhost Research Databases	1,146
		Search modes - Boolean/Phrase	Search Screen - Advanced Search	
			Database - CINAHL Complete	
S26	""patient communication behavior""	Expanders - Apply equivalent subjects	Interface - EBSCOhost Research Databases	16
		Search modes - Boolean/Phrase	Search Screen - Advanced Search	
			Database - CINAHL Complete	
S25	""physician decision making""	Expanders - Apply equivalent subjects	Interface - EBSCOhost Research Databases	304

Supplementary Material 1

		Search modes - Boolean/Phrase	Search Screen - Advanced Search	
			Database - CINAHL Complete	
S24	""patient-physician decision making""	Expanders - Apply equivalent subjects	Interface - EBSCOhost Research Databases	15
		Search modes - Boolean/Phrase	Search Screen - Advanced Search	
			Database - CINAHL Complete	
S23	""communication patient-clinician""	Expanders - Apply equivalent subjects	Interface - EBSCOhost Research Databases	2
		Search modes - Boolean/Phrase	Search Screen - Advanced Search	
			Database - CINAHL Complete	
S22	""communication patient-nurse""	Expanders - Apply equivalent subjects	Interface - EBSCOhost Research Databases	1
		Search modes - Boolean/Phrase	Search Screen - Advanced Search	
			Database - CINAHL Complete	
S21	""communication patient-doctor""	Expanders - Apply equivalent subjects	Interface - EBSCOhost Research Databases	1
		Search modes - Boolean/Phrase	Search Screen - Advanced Search	
			Database - CINAHL Complete	
S20	""patient-centred communication""	Expanders - Apply equivalent subjects	Interface - EBSCOhost Research Databases	112

Supplementary Material 1

		Search modes - Boolean/Phrase	Search Screen - Advanced Search	
			Database - CINAHL Complete	
S19	""patient-centered communication""	Expanders - Apply equivalent subjects	Interface - EBSCOhost Research Databases	447
		Search modes - Boolean/Phrase	Search Screen - Advanced Search	
			Database - CINAHL Complete	
S18	""patient centredness""	Expanders - Apply equivalent subjects	Interface - EBSCOhost Research Databases	347
		Search modes - Boolean/Phrase	Search Screen - Advanced Search	
			Database - CINAHL Complete	
S17	""patient-centeredness""	Expanders - Apply equivalent subjects	Interface - EBSCOhost Research Databases	747
		Search modes - Boolean/Phrase	Search Screen - Advanced Search	
			Database - CINAHL Complete	
S16	""client centered therapy""	Expanders - Apply equivalent subjects	Interface - EBSCOhost Research Databases	32
		Search modes - Boolean/Phrase	Search Screen - Advanced Search	
			Database - CINAHL Complete	
S15	""client participation""	Expanders - Apply equivalent subjects	Interface - EBSCOhost Research Databases	171

Supplementary Material 1

		Search modes - Boolean/Phrase	Search Screen - Advanced Search	
			Database - CINAHL Complete	
S14	""client satisfaction""	Expanders - Apply equivalent subjects	Interface - EBSCOhost Research Databases	900
		Search modes - Boolean/Phrase	Search Screen - Advanced Search	
			Database - CINAHL Complete	
S13	""therapeutic processes""	Expanders - Apply equivalent subjects	Interface - EBSCOhost Research Databases	1,126
		Search modes - Boolean/Phrase	Search Screen - Advanced Search	
			Database - CINAHL Complete	
S12	""personali?ed medicine""	Expanders - Apply equivalent subjects	Interface - EBSCOhost Research Databases	2,87
		Search modes - Boolean/Phrase	Search Screen - Advanced Search	
			Database - CINAHL Complete	
S11	""doctor-patient relations*""	Expanders - Apply equivalent subjects	Interface - EBSCOhost Research Databases	17,844
		Search modes - Boolean/Phrase	Search Screen - Advanced Search	
			Database - CINAHL Complete	
S10	(MH "Nurse-Patient Relations") OR "nurse-patient relations*"	Expanders - Apply equivalent subjects	Interface - EBSCOhost Research Databases	30,246

Supplementary Material 1

		Search modes - Boolean/Phrase	Search Screen - Advanced Search	
			Database - CINAHL Complete	
S9	(MH "Professional-Patient Relations+") OR "Professional-Patient Relations"	Expanders - Apply equivalent subjects	Interface - EBSCOhost Research Databases	101,752
		Search modes - Boolean/Phrase	Search Screen - Advanced Search	
			Database - CINAHL Complete	
S8	""patient participation""	Expanders - Apply equivalent subjects	Interface - EBSCOhost Research Databases	14,249
		Search modes - Boolean/Phrase	Search Screen - Advanced Search	
			Database - CINAHL Complete	
S7	(MH "Decision Making+") OR "Decision Making"	Expanders - Apply equivalent subjects	Interface - EBSCOhost Research Databases	177,659
		Search modes - Boolean/Phrase	Search Screen - Advanced Search	
			Database - CINAHL Complete	
S6	(MH "Decision Making, Shared")	Expanders - Apply equivalent subjects	Interface - EBSCOhost Research Databases	2,546
		Search modes - Boolean/Phrase	Search Screen - Advanced Search	
			Database - CINAHL Complete	
S5	(MH "Patient Preference") OR "Patient Preference"	Expanders - Apply equivalent subjects	Interface - EBSCOhost Research Databases	3,26

		Search modes - Boolean/Phrase	Search Screen - Advanced Search	
			Database - CINAHL Complete	
S4	(MH "Patient Satisfaction+") OR "Patient Satisfaction"	Expanders - Apply equivalent subjects	Interface - EBSCOhost Research Databases	68,338
		Search modes - Boolean/Phrase	Search Screen - Advanced Search	
			Database - CINAHL Complete	
S3	(MH "Physician-Patient Relations") OR "Physician-Patient Relation"	Expanders - Apply equivalent subjects	Interface - EBSCOhost Research Databases	34,878
		Search modes - Boolean/Phrase	Search Screen - Advanced Search	
			Database - CINAHL Complete	
S2	""patient centred care""	Expanders - Apply equivalent subjects	Interface - EBSCOhost Research Databases	17,199
		Search modes - Boolean/Phrase	Search Screen - Advanced Search	
			Database - CINAHL Complete	
S1	(MH "Patient Centered Care") OR "Patient Centered Care"	Expanders - Apply equivalent subjects	Interface - EBSCOhost Research Databases	34,989
		Search modes - Boolean/Phrase	Search Screen - Advanced Search	
			Database - CINAHL Complete	

Search #5 - #11

Scandinavian databases

Supplementary Material 1

Database #	Database	Search terms
#5	Oria	(barn OR ungdom OR tenåringer OR unge voksne) AND (psykisk OR psykologi OR psykiatri OR BUP OR helse* OR mental OR barnepsykologi OR barnepsykiatri OR ungdomspsykologi OR ungdomspsykiatri OR helsetjenester) AND (metode OR metoder OR instrument OR verktøy OR modell OR modeller OR kulturformidling OR intervju* OR spørreskjema OR kartlegging) AND (brukermedvirkning OR brukererfaringer OR brukererfaring OR erfaringer OR erfaring OR opplevelse OR opplevelser OR samspill OR allianse* OR samhandling OR pasientsentrert OR pasient-sentrert OR pasientrettet OR medbestemmelse OR relasjon OR relasjoner OR pasientmedvirkning OR samvalg OR pasientkommunikasjon OR PCC OR personsentert OR person-sentrert OR empowerment)
#6	Idunn	#6 a)[1]<br>(barn* OR ungdom* OR tenåring* OR unge voksne) AND (psyk* OR BUP OR helse* OR mental OR barnepsyk* OR ungdomspsyk* OR helsetjeneste*) AND (brukermedvirkning OR brukererfaring* OR erfaring* OR opplevelse* OR samspill OR allianse* OR samhandling OR pasientsentrert OR pasientrettet OR medbestemmelse OR relasjon* OR pasientmedvirkning OR medvirkning OR samvalg OR pasientkommunikasjon) AND (metode OR metoder OR instrument OR verktøy OR modell OR modeller OR kulturformidling OR intervju* OR spørreskjema OR kartlegging)<br><br>#6 b)*<br>(barn* OR ungdom* OR tenåring* OR unge voksne) AND (psyk* OR BUP OR helse* OR mental OR barnepsyk* OR ungdomspsyk* OR helsetjeneste*) AND (personsentert OR empowerment OR PCC) AND (metode OR metoder OR instrument OR verktøy OR modell OR modeller OR kulturformidling OR intervju* OR spørreskjema OR kartlegging)
#7	Norart	(barn* OR ungdom*) AND (psyk* OR bup OR mental) AND (brukermedvirkning OR allianse* OR samhandling OR pasient*sentrert OR pasient*rettet OR medbestemmelse OR relasjon* OR pasient*medvirkning OR samvalg OR pcc OR person*sentrert)
#8	Svemed+	1 Barnpsykiatri 343<br>2 Barnpsykologi 265<br>3 Ungdomspsykiatri 272<br>4 Ungdomspsykologi 218<br>5 1 OR 2 OR 3 OR 4 794<br>6 barn 13282<br>7 Barns hälsa 848<br>8 Barnhälsovård 544<br>9 Ungdomar 9938<br>10 Ungdomars hälsa 727<br>11 Unga vuxna 2638<br>12 6 OR 7 OR 8 OR 9 OR 10 OR 11 19539<br>13 Psykiatri 3210<br>14 Psykologi 2403<br>15 Psykiatrisk vård 2320<br>16 13 OR 14 OR 15 7128

[1] Search in Idunn had to be conducted in two separate searches, due to limitations in the search interface

Supplementary Material 1

		17 12 AND 16 1596<br>18 5 OR 17 1643<br>19 Patientcentrerad vård 615<br>20 Beslutsfattande 2113<br>21 Delat beslutfattande 1<br>22 Personcentrerad psykoterapi 1<br>23 Läkare-patientrelationer 1859<br>24 Patienttillfredsställelse 1701<br>25 Patientpreferens 55<br>26 Patientmedverkan 818<br>27 Hälsopersonal-patientrelationer 4879<br>28 Sjuksköterska-patientrelationer 1455<br>29 relationer 957<br>30 19 OR 20 OR 21 OR 22 OR 23 OR 24 OR 25 OR 26 OR 27 OR 28 OR 29 9727<br>31 18 AND 30 184
#9	Publicera.se	(Barn* OR Ungdom*) (Barnpsyk* OR Ungdomspsyk* OR Psyk* OR Mental*) (Patientcentrerad OR Personcentrerad OR Patienttillfreds* OR Patientpreferens* OR Patientmedverkan OR Beslutsfattande OR medverkan)
#10	Swepub	(Barn* OR Ungdom*) (Barnpsyk* OR Ungdomspsyk* OR Psyk* OR Mental*) (Patientcentrerad OR Personcentrerad OR Patienttillfreds* OR Patientpreferens* OR Patientmedverkan OR Beslutsfattande OR medverkan)
#11	Tidsskrift.dk	Boolean operators not applicable. Search terms used:<br>Børn<br>Ungdom<br>Teenager<br><br>Psykologi<br>Psykiatri<br><br>Brugerdeltagelse<br>Brugeroplevelser<br>Erfaring<br>Oplevelse<br>Interaktion<br>Alliance<br>Patientcentreret<br>Patientorienteret<br>Medbestemmelse<br>Samvalg<br>Patientkommunikation<br>PCC<br>Personcentreret<br>Empowerment<br>Brugerinddragelse
