# Supplementary material for: Adolescents’ voices on self-engagement in mental health treatment: a scoping review
Source: Eur Child Adolesc Psychiatry. 2024 Mar 27;33(12):4083–95. doi: 10.1007/s00787-024-02425-7 (PMC11618195; doi:10.1007/s00787-024-02425-7)
Supplement: Supplementary file 2 — Supplementary Material 2 [file 787_2024_2425_MOESM2_ESM.pdf]

Supplementary Material 2

Critical Appraisal Skills Programme (CASP) results

Adolescents' voices on self-engagement in mental health treatment: a scoping review

European Child & Adolescent Psychiatry

Nina Therese Øversveen Svamo, Research Center for Existential Health, Innlandet Hospital Trust, Norway; Inland Norway University of Applied Sciences Lillehammer, Nina.Therese.Oversveen.Svamo@sykehuset-innlandet.no

Sigrid Helene Kjørven Haug

Valerie DeMarinis

Urd Hertzberg

Reference	1	2	3	4	5	6	7	8	9	10	Score
Armstrong et al. (2019)	Y	Y	C	Y	Y	Y	Y	Y	Y	Y	High (9)
Buston (2002	Y	Y	C	Y	Y	Y	Y	C	Y	Y	Moderate (8)
Byczkowski et al. (2010)	Y	Y	C	Y	Y	N	Y	Y	Y	Y	Moderate (8)
Coates (2016)	Y	Y	C	Y	Y	Y	Y	Y	Y	Y	High (9)
Coyne et al. (2015)	Y	Y	Y	Y	Y	N	Y	Y	Y	Y	High (9)
Davison et al. (2017)	Y	Y	Y	Y	Y	N	Y	Y	Y	Y	High (9)
Grealish et al. (2013)	Y	Y	Y	Y	Y	Y	Y	Y	Y	Y	High (10)
Harper et al. (2014)	Y	Y	Y	Y	Y	N	Y	Y	Y	Y	High (9)
Hart et al. (2005)	Y	Y	Y	Y	Y	N	Y	C	Y	Y	Moderate (8)
Hayes et al. (2020)	Y	Y	Y	Y	Y	Y	Y	Y	Y	Y	High (10)
Jones et al. (2017)	Y	Y	Y	Y	Y	Y	Y	Y	Y	Y	High (10)
LeFrançois (2008)	Y	Y	Y	C	Y	C	N	C	Y	Y	Moderate (6)
McCann and Lubman (2012)	Y	Y	Y	Y	Y	N	Y	Y	Y	Y	High (9)
Midgley et al. (2016)	Y	Y	Y	Y	Y	Y	Y	Y	Y	Y	High (10)
Munford and Sanders (2016)	Y	Y	C	Y	Y	Y	N	Y	Y	Y	Moderate (8)
Persson et al. (2017)	Y	Y	Y	Y	Y	N	Y	Y	Y	Y	High (9)
Ronzoni and Dogra (2012)	Y	Y	Y	Y	Y	N	N	Y	Y	Y	Moderate (8)
Salamone-Violi et al. (2015)	Y	Y	Y	Y	Y	Y	Y	Y	Y	Y	High (10)
Stafford et al. (2016)	Y	Y	C	Y	Y	N	Y	Y	Y	Y	Moderate (8)

Y = Yes – identified in the study, N = No – not identified in the study, C = Can`t tell – authors not clear/not specified.

Supplementary Material 2

CASP Qualitative Studies Checklist consists of ten criteria on study design: 1. Was there a clear statement of the aims of the research?; 2. Was a qualitative methodology appropriate?; 3. Was the research design appropriate to address the aims of the research?; 4. Was the recruitment strategy appropriate to the aims of the research?; 5. Was the data collected in a way that addressed the research issue?; 6. Has the relationship between researcher and participants been adequately considered?; 7 Have ethical issues been considered?; 8. Was the data analysis sufficiently rigorous?; 9. Is there a clear statement of the findings?; 10. How valuable is the research? (Critical Appraisal Skills Programme, 2018)

Score: A grading system was used where studies received 1 point for each criterion met. Methodological quality was graded as low when meeting 0-5 criteria, moderate when meeting 6–8 criteria and high with 9–10 criteria of the CASP checklist. For question 10, the score was considered to be Yes if the study was considered to be relevance to the scoping review.
